# Breath Ketone Testing: A New Biomarker for Diagnosis and Therapeutic Monitoring of Diabetic Ketosis

**DOI:** 10.1155/2014/869186

**Published:** 2014-05-11

**Authors:** Yue Qiao, Zhaohua Gao, Yong Liu, Yan Cheng, Mengxiao Yu, Lingling Zhao, Yixiang Duan, Yu Liu

**Affiliations:** ^1^Department of Endocrinology, The Second Hospital of Jilin University, 218 Ziqiang Road, Changchun, Jilin 130041, China; ^2^Research Center of Analytical Instrumentation, Analytical & Testing Center, Sichuan University, 29 Wangjiang Road, Chengdu 610064, China

## Abstract

*Background*. Acetone, **β**-hydroxybutyric acid, and acetoacetic acid are three types of ketone body that may be found in the breath, blood, and urine. Detecting altered concentrations of ketones in the breath, blood, and urine is crucial for the diagnosis and treatment of diabetic ketosis. The aim of this study was to evaluate the advantages of different detection methods for ketones, and to establish whether detection of the concentration of ketones in the breath is an effective and practical technique. *Methods*. We measured the concentrations of acetone in the breath using gas chromatography-mass spectrometry and **β**-hydroxybutyrate in fingertip blood collected from 99 patients with diabetes assigned to groups 1 (−), 2 (±), 3 (+), 4 (++), or 5 (+++) according to urinary ketone concentrations. *Results*. There were strong relationships between fasting blood glucose, age, and diabetic ketosis. Exhaled acetone concentration significantly correlated with concentrations of fasting blood glucose, ketones in the blood and urine, LDL-C, creatinine, and blood urea nitrogen. *Conclusions*. Breath testing for ketones has a high sensitivity and specificity and appears to be a noninvasive, convenient, and repeatable method for the diagnosis and therapeutic monitoring of diabetic ketosis.

## 1. Introduction

Diabetic ketoacidosis (DKA) is a life-threatening condition that occurs predominantly in patients with newly diagnosed type 1 diabetes mellitus and is a consequence of a lack of insulin production by pancreatic islet cells, but it may also occur in patients with type 2 diabetes with poorly controlled blood glucose concentration or other diseases [1]. Diabetic ketosis and ketoacidosis are mainly caused by a lack of insulin or an inappropriate rise in blood glucagon concentration that leads to sugar, protein, fat, water, electrolyte, and acid-base imbalance. Identifying a testing method with high sensitivity and specificity would facilitate the early diagnosis and treatment of diabetic ketosis.

Ketone bodies are produced when the liver metabolizes fatty acids, including acetone, *β*-hydroxybutyrate, and acetoacetic acid: *β*-hydroxybutyrate can be converted into acetoacetic acid and accounts for 78% of all ketones in the body, followed by acetoacetic acid (20%) and acetone (2%). Clinically, when making the diagnosis of DKA, blood ketone concentration is generally inferred from the urinary ketone concentration. Commonly used detection methods for urinary ketones are more sensitive to acetoacetic acid than acetone but less sensitive to *β*-hydroxybutyrate, which appears earliest in DKA—explaining why patients with DKA may not have detectable concentrations of ketones in their urine. Urinary ketone excretion may also be impaired in patients with renal dysfunction. It can be argued that detecting urinary ketones is not a suitable means of diagnosing DKA.

A blood test that measures the concentration of serum *β*-hydroxybutyrate is available, but there has been a great deal of interest in developing means of measuring the concentration of ketones in the breath, as a convenient and noninvasive diagnostic tool that could also guide therapeutic interventions. The presence of acetone in the breath has long been known to be correlated with ketone bodies in the plasma. Acetoacetate may be decarboxylated to produce volatile acetone, besides that the boiling point of acetoacetate and *β*-hydroxybutyric acid in exhaled breath is higher than acetone, with the content relatively small and difficult to detect, so we choose the acetone concentrations as a predictor of diabetic ketosis. We evaluated the advantages of various detection methods and explored the clinical value of acetone breath detection in the diagnosis and treatment of diabetic ketosis.

## 2. Materials and Methods

### 2.1. Participants

Ninety-nine patients with diabetes (49 males and 50 females; age range: 11–85 years) were recruited from the Department of Endocrinology of the Second Hospital of Jilin University in Changchun, China. According to the urinary ketone detecting package insert, color changes of –, ±, +, ++, and +++ correspond to concentrations of 0 mmol/L, 0.5 mmol/L, 1.5 mmol/L, 3.9 mmol/L, and 7.8 mmol/L, respectively. The patients were assigned into 5 groups on the basis of urinary ketone concentration: group 1 (−), urinary ketone recorded as negative, 9 males and 10 females (*n* = 19); group 2 (±), urinary ketone recorded as mild positive, 7 males and 9 females (*n* = 16); group 3 (+), urinary ketone recorded as positive, 14 males and 11 females (*n* = 25); group 4 (++), urinary ketone recorded as moderate positive, 9 males and 10 females (*n* = 19); and group 5 (+++), urinary ketone recorded as strong positive, 10 males and 10 females (*n* = 20). The study protocol was approved by the Ethics Committee of the Second Hospital of Jilin University, and written consent was obtained from all subjects before breath collection.

### 2.2. Inclusion Criteria

Type 2 diabetes mellitus was diagnosed according to the 1999 WHO diagnostic criteria [2]. Patients with gestational diabetes, diabetes mellitus complicating pregnancy, and secondary diabetes were excluded.

### 2.3. Measurement of Ketone Concentration

Fresh fingertip blood samples were obtained and the blood concentration of *β*-hydroxybutyrate was measured using an Optium Xceed (Abbott, USA) device: using the manufacturer-suggested cutoff of >0.5 mmol/L was considered to be positive. We used 3 L foil bags to collect exhaled breath from participants, which were analyzed within 5 days. Three samples of exhaled breath were obtained from each subject. The concentration of acetone was determined in the breath using gas chromatography-mass spectrometry (GC/MS). Operation was performed according to the instructions. Quality control of the exhaled breath has been described in our published paper [3]. A concentration ≥1.0 ppmv was considered positive. Urinary ketone concentrations were also measured, and the demographic and clinical characteristics of patients were recorded.

### 2.4. Statistical Analysis

All data were statistically processed using SPSS software (version 17; IBM, New York, NY, USA) and reported as mean ± standard deviation (SD). Intergroup comparisons were performed using *t*-tests for normally distributed data and nonparametric tests for data that were not normally distributed. Analysis of variance was used for multigroup comparisons. Categorical data were analyzed using chi-square tests and expressed as positive cases and constituent ratios (%). Correlation analysis was performed to examine the strength of relationships between variables. A receiver operating characteristic (ROC) curve was constructed to determine the optimal cut-off value of concentration of exhaled acetone and the urinary ketone, and sensitivity and specificity were calculated. Two-sided tests were used for all statistical analyses. A *P* value < 0.05 was considered statistically significant.

## 3. Results

### 3.1. Demographic and Clinical Characteristics of Participants

Demographic and clinical data are shown in Table 1. Fasting blood glucose (FBG) concentration on admission was significantly higher in group 5 than groups 1, 2, 3, and 4 (*P* < 0.001, *P* = 0.005, *P* = 0.029, and *P* = 0.008, resp.), but there were no differences between groups 1 to 4. Patients in group 5 were significantly younger than those in groups 1 to 3 (*P* = 0.005, *P* = 0.001, and *P* = 0.001, resp.), and patients in group 4 were also younger than those in group 2 (*P* = 0.037), but there were no statistically significant differences in age between the other groups. Furthermore, there were no significant differences in sex, body mass index, blood hemoglobin A1c (HbA1c), total cholesterol (TC), triglyceride (TG), low-density lipoprotein cholesterol (LDL-C), high-density lipoprotein cholesterol (HDL-C), aspartate aminotransferase (AST), alanine aminotransferase (ALT), creatinine (Cr), and blood urea nitrogen (BUN) concentration between any of the five groups.

### 3.2. Comparison of Blood and Breath Concentrations of Ketones

**Figure 1:**
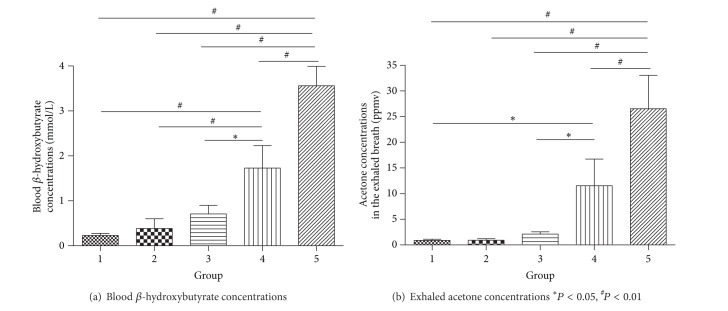
Blood *β*-hydroxybutyrate and exhaled acetone concentrations in patients with increasing concentrations of urinary ketones.

Concentrations of blood *β*-hydroxybutyrate and exhaled acetone are shown in Table 2 and Figure 1. The blood concentration of *β*-hydroxybutyrate was significantly higher in groups 4 and 5 than groups 1 to 3 (*P* = 0.003, *P* = 0.008, and *P* = 0.023, resp., and *P* < 0.001, *P* < 0.001, and *P* < 0.001, resp.) and higher in group 5 than group 4 (*P* < 0.001), but there were no differences between groups 1 to 3. The breath concentration of acetone was higher in group 4 than groups 1 and 3 (*P* = 0.028 and *P* = 0.035, resp.) and higher in group 5 than groups 1 to 4 (*P* < 0.001, *P* < 0.001, *P* < 0.001, and *P* = 0.002, resp.), but there were no differences between the other groups. Blood *β*-hydroxybutyrate concentration was positive in 6.7%, 14.3%, 43.5%, 71.4%, and 89.5% of cases, respectively, in groups 1 to 5, and exhaled acetone concentration was positive in 18.8%, 20%, 60%, 80%, and 92.9% of cases, respectively, in groups 1 to 5 (Table 3).

### 3.3. Correlation of Urinary Ketone Concentration with Exhaled Breath Acetone

The exhaled acetone concentration was significantly correlated with the concentrations of FBG (*r* = 0.428, *P* < 0.001), blood *β*-hydroxybutyrate (*r* = 0.817, *P* < 0.001), urinary ketone concentration (*r* = 0.581, *P* < 0.001), LDL-C (*r* = 0.255, *P* = 0.047), Cr (*r* = 0.385, *P* = 0.002), and BUN (*r* = 0.362, *P* = 0.003) (Table 4).

### 3.4. Exhaled Acetone Concentration as a Predictor of Diabetic Ketosis

**Figure 2:**
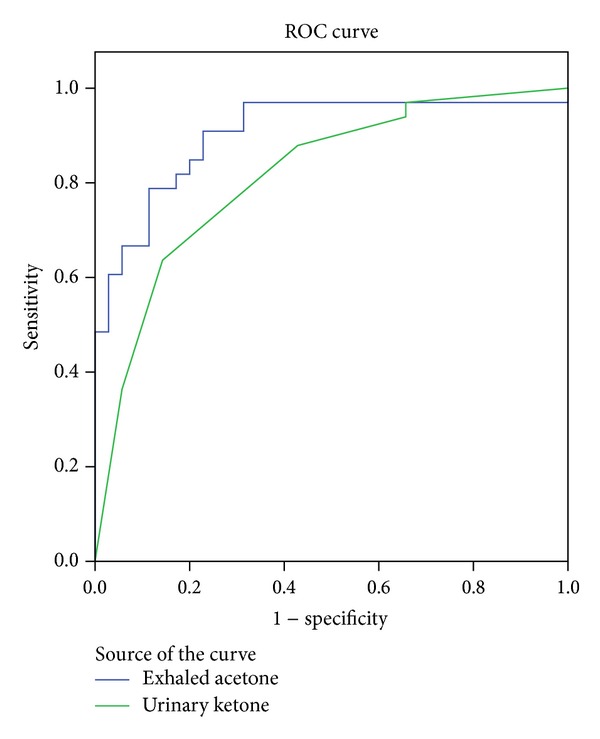
Receiver operating characteristic (ROC) curve for exhaled acetone and urinary ketone concentration for the diagnosis of diabetic ketosis.

Concentrations of blood *β*-hydroxybutyrate served as the standard to assess the sensitivity and specificity of exhaled acetone for detection of diabetic ketosis (Figure 2). The area under the curve (AUC) was 0.905 (*P* < 0.001), and the cut-off concentration of exhaled acetone for diagnosis of diabetic ketosis was 1.185 ppmv, with a sensitivity and specificity of 90.9% and 77.1%, respectively. Concentrations of blood *β*-hydroxybutyrate served as the standard to assess the sensitivity and specificity of urinary ketone for detection of diabetic ketosis (Figure 2). The area under the curve (AUC) was 0.815 (*P* < 0.001), and the cut-off concentration of urinary ketone for diagnosis of diabetic ketosis was 2.7 mmol/L, with a sensitivity and specificity of 63.6% and 85.7%, respectively.

## 4. Discussion

Ketoacidosis may occur in patients with diabetes of all ages [4]. A study of Austrian indicated that the incidence of DKA was negatively correlated with age [5]. Klingensmith and colleagues have reported that younger age, lack of private health insurance, and African American ancestral heritage are independent risk factors for DKA [6]. In our study, younger patients and higher FBG concentration tended to be strongly positive for urinary ketones, which is consistent with data reported.

Exhaled breath detection has been used to diagnose metabolic disease and monitor treatment for many years [7]. The techniques used to detect these compounds in exhaled breath are based on mass spectrometry, for example, proton transfer reaction mass spectrometry, selected ion flow tube mass spectrometry [8], and cavity ring down spectroscopy. The concentration of breath acetone is associated with glucose metabolism and lipolysis [7]. Previous studies have shown a close correlation between the concentrations of ketones released from the skin and blood levels [9]. Breath acetone concentration is also reported to be elevated in type 2 diabetes mellitus, and it can be used to diagnose the onset of diabetes [10]. We used the GC/MS method to detect exhaled acetone, which is capable of detecting over 200 constituents of exhaled breath and is highly sensitive to typical volatile organic compounds. In our study correlation analysis demonstrated that the concentration of exhaled acetone was significantly associated with urinary ketone concentration, blood FBG, LDL-C, Cr, and BUN concentrations. Prompt exhaled acetone maybe is a better index in reflecting the changes of blood glucose, and testing for exhaled acetone is a noninvasive, simple method, which is expected to be a promising indicator of blood glucose monitoring in the future.

When the concentration of blood *β*-hydroxybutyrate served as the standard in our study to assess the sensitivity and specificity of exhaled acetone and urine ketone, the sensitivity and specificity of exhaled acetone were 90.9% and 77.1%, respectively. However, the sensitivity and specificity of urine ketone were 63.6% and 85.7%, respectively. These results show that the specificity of exhaled acetone is similar to urine ketone, but its sensitivity is higher than urine ketones. In addition, the testing for blood *β*-hydroxybutyrate and the exhaled acetone is still positive in the urine ketone body negative group; the proportion is 6.7% and 18.8%, respectively. So the concentration of urine ketones may not be a timely predictor of early diabetic ketosis. Blood and exhaled testing for ketones helps to eliminate false negative results [11]. Another potential value for breath ketones testing is it being strongly influenced by physiological factors other than diet [3]. In the present method, the concentration of exhaled acetone higher than 1.185 ppmv was found in diabetic ketosis patients; the detection just needs simple preparation and no organic solvent. Exhaled acetone analysis proves to be a noninvasive, convenient, sensitive, and solvent-free method and could be applied to diagnose and monitor the severity of diabetic ketosis. However, the technique is still preliminary and its wide clinical use requires further optimization.

## Figures and Tables

**Table 1:** Demographic and clinical characteristics of study participants.

	1 Urine ketone (−)	2 Urine ketone (±)	3 Urine ketone (+)	4 Urine ketone (++)	5 Urine ketone (+++)	*P *
Age (yr)	45	48	45	37	30	0.004
Male (*n*)	9 (18.37%)	7 (14.29%)	14 (28.57%)	9 (18.37%)	10 (20.40%)	0.783
BMI (kg/m^2^)	23.49	25.41	22.73	23.43	21.92	0.219
FBG (mmol/L)	13.27	14.36	16.29	15.08	20.36	0.006
HbA1c (%)	10.37	10.59	11.40	10.33	12.26	0.183
TC (mmol/L)	5.78	5.27	6.06	5.01	5.62	0.327
TG (mmol/L)	2.71	3.39	3.60	1.47	4.83	0.439
LDL-C (mmol/L)	3.08	3.04	3.02	2.86	3.02	0.99
HDL-C (mmol/L)	1.12	1.10	1.24	1.20	1.12	0.747
ALT (U/L)	31.35	31.45	23.47	22.69	23.31	0.833
AST (U/L)	23.65	24.18	25.04	25.36	18.94	0.830
BUN (mmol/L)	3.84	4.20	4.05	4.58	4.46	0.745
Cr (*μ*mol/L)	58.06	64.16	61.66	62.13	70.32	0.584

BMI: body mass index; FBG: fasting blood glucose; HbA1c: hemoglobin A1c; TC: total cholesterol; TG: triglyceride; LDL-C: low-density lipoprotein cholesterol; HDL-C: high-density lipoprotein cholesterol; AST: aspartate aminotransferase; ALT: alanine aminotransferase; Cr: creatinine; BUN: blood urea nitrogen.

**Table 2:** Comparison of blood *β*-hydroxybutyrate concentrations and exhaled acetone concentrations between the groups.

	1 Urine ketone (−)	2 Urine ketone (±)	3 Urine ketone (+)	4 Urine ketone (++)	5 Urine ketone (+++)	*P *
Blood *β*-hydroxybutyrate (mmol/L)	0.23	0.39	0.71	1.73	3.56	<0.001
Acetone in the breath (ppmv)	0.89	0.93	2.04	13.82	33.12	<0.001

**Table 3:** Incidence of positive blood *β*-hydroxybutyrate and exhaled acetone detection in each group.

	1 Urine ketone (−)	2 Urine ketone (±)	3 Urine ketone (+)	4 Urine ketone (++)	5 Urine ketone (+++)	*P *
Blood *β*-hydroxybutyrate	6.7%	14.3%	43.5%	71.4%	89.5%	<0.001
Acetone in the breath	18.8%	20%	60%	80%	92.9%	<0.001

**Table 4:** Correlation between exhaled acetone concentration and other clinical variables.

	Correlation coefficient (*r*)	*P *
FBG	0.428	<0.001
Blood *β*-hydroxybutyrate	0.817	<0.001
Urine ketone	0.581	<0.001
LDL-C	0.255	0.047
Cr	0.385	0.002
BUN	0.362	0.003

FBG: fasting blood glucose; LDL-C: low-density lipoprotein cholesterol; Cr: creatinine; BUN: blood urea nitrogen.
